# A 16-gene signature associated with homologous recombination deficiency for prognosis prediction in patients with triple-negative breast cancer

**DOI:** 10.1515/med-2022-0475

**Published:** 2022-05-11

**Authors:** Daodu Wang, Yifeng Shi, Hanyang Huang, Qijiong Zhao, Yongyue He, Wenzhi Su

**Affiliations:** Oncology Center, Shanwei Yihui Fund Hospital (Shanwei Second People’s Hospital), Shanwei, 516600, China; Department of General Surgery, Shanwei Yihui Fund Hospital (Shanwei Second People’s Hospital), Shanwei, 516600, China

**Keywords:** breast cancer, triple-negative, prognosis model, homologous recombination deficiency, HRD score

## Abstract

Homologous recombination deficiency (HRD) commonly occurs in breast cancer, which is the second cause of cancer death in women with a high rate of relapse and poor outcomes. Triple-negative breast cancer (TNBC) is the most aggressive subtype of breast cancer. Thus, we aim to develop a prognostic signature based on HRD expecting to help improve outcomes in TNBC. The Cancer Genome Atlas (TCGA)–TNBC cohort was divided into the training set and the testing set randomly. Sixteen genes were filtered from the prognostic HRD-associated genes to establish a prognostic model in the training set. Patients were divided into high-risk and low-risk groups based on the median value of the risk score. Prognosis analysis showed that the high-risk group was associated with a worse prognosis in the training set, the testing set, the entire TCGA–TNBC cohort, and the METABRIC–TNBC cohort. The time-dependent receiver operating characteristic curve showed that our model had very good accuracy in the prediction of 1–5-year overall survival in the TCGA–TNBC cohort. Besides, a comparison of the area under curve value and *C*-index between our model and four published models showed that our model had the best predictive efficiency compared to other models. Subsequently, a nomogram was established. Finally, our finding also indicated that our model was associated with immunoregulation in TNBC and had the potential to be the target for TNBC treatment. Therefore, our findings not only provided a new strategy in the personalized prognosis management of TNBC but also offered new insight into precision treatment in TNBC.

## 1 Introduction

Breast cancer is the most frequently diagnosed cancer in women. Breast cancer is also the second cause of cancer death in women because of its highly heterogeneous and complex biological features [1]. Typically, breast-conserving surgery and radiation therapy are the main therapies for patients with early-stage breast cancer [2] and the 5-year survival rate of early-stage patients is respectable. However, despite the successful achievement of targeted therapy and immunotherapy in breast cancer, the prognosis of patients with recurrent or metastatic breast cancer is still very poor [3]. Hence, noninvasive biomarkers that are useful in the diagnosis and prognosis of breast cancer would be of significant benefit for breast cancer management. Triple-negative breast cancer (TNBC), a specific subtype of breast cancer that does not express estrogen receptor, progesterone receptor, or human epidermal growth factor receptor 2 (HER-2) [4,5], is not sensitive to endocrine therapy or HER-2-targeted therapy. Therefore, TNBC is also a particularly aggressive subtype of breast cancer with high invasiveness, high metastatic potential, proneness to relapse, and poor prognosis. Besides, standardized treatment regimens for patients with TNBC are still lacking. Hence, it is urgently needed to develop new therapeutic strategies.

Homologous recombination repair and poly-ADP-ribose polymerase (PARP) play important role in DNA double-strand break repair and apoptosis. Homologous recombination deficiency (HRD) is a frequent driver of tumorigenesis, and it most frequently occurred in breast cancer [6]. Breast cancer (BRCA) mutation is the most common cause of HRD, and germline BRCA mutations occur in 10–20% of patients with TNBC [7]. Therefore, DNA-damaging therapeutics, such as PARP inhibitors, have been a new strategy for TNBC treatment [8]. HRD score is an unweighted sum of loss of heterozygosity [9], telomeric allelic imbalance [10], and large-scale state transitions [11] scores, which is developed to measure genomic instability in tumors [12,13]. Recently, HRD score has become a new stratification for patients with TNBC. Patients with high-HRD score are more likely to respond to PARP inhibitors and platinum-containing therapy [14,15].

In this study, two TNBC cohorts from The Cancer Genome Atlas (TCGA) and the molecular taxonomy of breast cancer international consortium (METABRIC) databases were enrolled. We first divided the TCGA–TNBC cohort into high HRD (HRD score >41) and low HRD (HRD score ≤41) and filtered the differentially expressed genes between two groups. And then, we established the HRD-associated genes prognostic model in TNBC by the LASSO Cox method and tested the accuracy and independence of the model. Subsequently, we compared the predictive efficiency of the model and other established models. Finally, we preliminarily unveiled the potential mechanism of the model in TNBC. Our study attempted to provide a new strategy for risk stratification in TNBC and expected to offer new thoughts for precision treatment in TNBC.

## 2 Methods

### 2.1 Data collection

The transcriptome profile and the clinicopathologic data of patients with TNBC were downloaded from the TCGA database (https://portal.gdc.cancer.gov). The somatic mutation counts and copy number variation were obtained from the cBioPortal database (http://www.cbioportal.org/study?id=brca_tcga_pan_can_atlas_2018). As validation, patients with TNBC in the METABRIC were employed and the transcriptome profile and clinical data were downloaded from the METABRIC database (http://molonc.bccrc.ca/aparicio-lab/research/metabric/). Eliminating the patients without survival information, a total of 460 patients with TNBC were enrolled in this study.

### 2.2 Identification of prognostic HRD-associated genes in TNBC

The HRD score of patients with TNBC in the TCGA cohort was calculated as described in previously published studies [12,14,16], and the patients with TNBC were divided into the high-HRD group and the low-HRD group according to the criteria of HRD score = 41. Differentially expressed gene (DEG) analysis was performed between two groups by using DESeq2 [17], and the filter criteria are as follows: false discovery rate (FDR) <0.05 and |fold change (FC)| ≥1.5. The DEGs were identified as the HRD-associated genes in TNBC. And then, the univariate analysis-Cox method was performed in the TCGA–TNBC cohort based on the HRD-associated genes and the genes that were associated with prognosis (*P*-value <0.05) were identified as the prognostic HRD-associated genes in TNBC. The selected prognostic HRD-associated genes were candidates for model establishment.

### 2.3 Model construction

One hundred and sixty-one patients with TNBC in the TCGA cohort were randomized into two groups (the training set and the testing set). Least absolute shrinkage and selection operator (LASSO) is a statistical formula for the regularization of data model and feature selection [18,19]. We used the LASSO Cox regression method to examine the relationship between prognostic HRD-associated genes and to subsequently identify the most relevant genes associated with prognosis in the training set. Subsequently, a signature based on the prognostic HRD-associated genes was established by the following formula:

\text{risk score}=\mathop{\sum }\limits_{i=1}^{n}\text{coefi}\hspace{.25em}\ast \hspace{.25em}\text{expri},]

where “coefi” represents coefficient of each gene in the model and “expri” represents the expression level of each selected gene.

### 2.4 Evaluation and validation of the model

The risk scores of each patient were calculated by the unified formula. To evaluate the prognostic value of the model, patients with TNBC in the training set were further divided into high-risk and low-risk groups based on the median value of the risk score. Kaplan–Meier survival curves with the log-rank test were used to examine the significant difference of overall survival (OS) between the two groups. As internal validation, the same process was conducted in the testing set and the entire TCGA–TNBC cohort. As external validation, 299 patients with TNBC in the METABRIC cohort were divided into two groups (high risk and low risk) based on the median value of the risk score. Kaplan–Meier survival analysis was used to validate the prognostic value of the model in the METABRIC–TNBC cohort.

To test the accuracy and independence of the model, receiver operating characteristic (ROC) analysis and multivariable analysis were performed in the TCGA–TNBC cohort. The value of area under curve (AUC) was used to evaluate the predictive efficiency of the model in 1-, 2-, 3-, 4-, and 5-year survival in the TCGA–TNBC cohort. And then, ROC curve analysis and accordance index (*C*-index) analysis were used to evaluate the predictive efficiency of our model and four recently reported models in 5-year survival were performed in the TCGA–TNBC cohort. Comparison of the predictive efficiency between our model and four recently reported models was performed according to the value of AUC and *C*-index of each model in the TCGA–TNBC cohort. Finally, a nomogram was constructed to support risk stratification clinically.

### 2.5 Potential mechanism exploration of the model in TNBC

One hundred and sixty-one patients with TNBC in the TCGA cohort were divided into high-risk and low-risk groups based on the median value of the risk scores. DESeq2 [17] was used to perform the DEG analysis between two groups, and the threshold value of differential gene analysis was FDR <0.05 and |FC| ≥1.5. Kyoto Encyclopedia of Genes and Genomes (KEGG) analyses [20] were conducted using R package “clusterProfiler” [21]. Gene Set Enrichment Analysis (GSEA) [22] was conducted using gseKEGG and gsePathway functions in R package “clusterProfiler” [21], with the parameters nPerm = 1,000, minGSSize = 10, maxGSSize = 1,000, and *P* value-Cutoff = 0.05.

### 2.6 Statistical analysis

Chi-squared and Mann–Whitney *U* tests were implemented to explore the differences in categorical and quantitative data between different datasets or groups, respectively. Statistical significance was defined when two-tailed *P* < 0.05. R version 4.0.2 (Institute for Statistics and Mathematics, Vienna, Austria) executed all the statistical analyses and visualization with the corresponding functional package.

## 3 Results

### 3.1 HRD-associated genes’ identification and model construction

**Figure 1:**
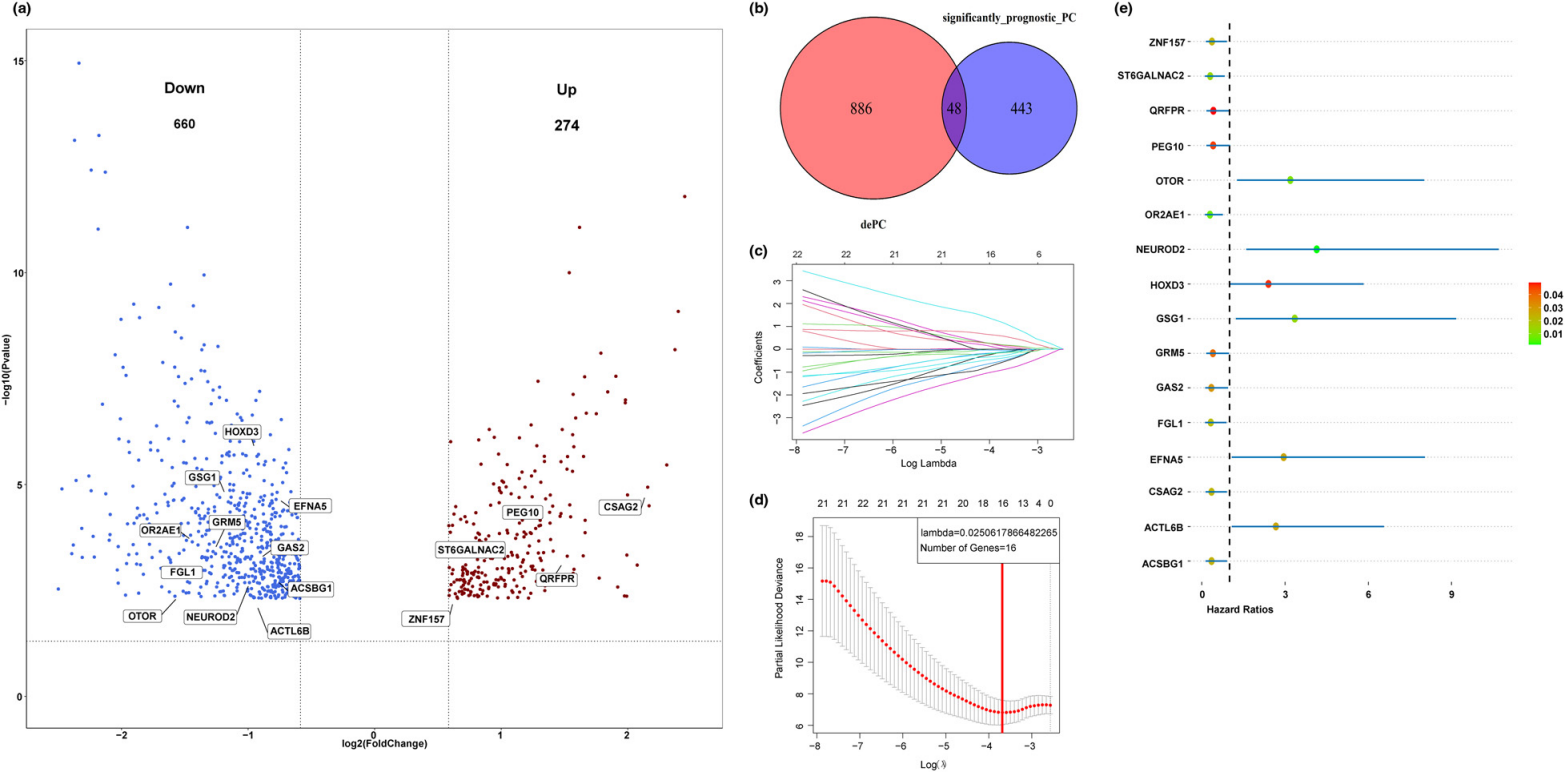
Identification of prognostic HRD-associated genes and model construction in the TCGA–TNBC cohort. (a) Volcano plot of DEGs between high-HRD and low-HRD groups. (b) Venn plot of prognostic HRD-associated genes. The red circle represents the DEGs and the blue circle represents the prognostic genes in TNBC. (c) The LASSO coefficient profile of 48 prognostic HRD-associated genes and perpendicular imaginary lines were drawn at the value chosen by 10-fold cross-validation. (d) The tuning parameters (log *l*) of OS-related proteins were selected to cross-verify the error curve. According to the minimal criterion and 1-se criterion, perpendicular imaginary lines were drawn at the optimal value. (e) Multivariate analysis of 16 selected genes in the TCGA–TNBC cohort.

**Figure A1:**
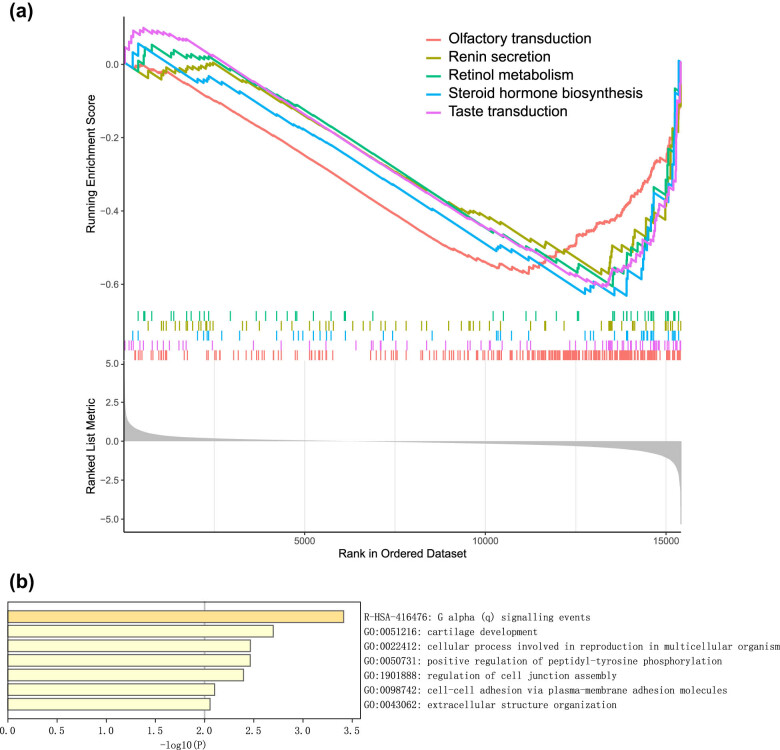
Heatmap of pathway enrichment analysis.

Patients with TNBC in the TCGA cohort were divided into two groups (high HRD and low HRD) based on the HRD score = 41. A total of 934 DEGs were identified, and among them, 660 DEGs were downregulated genes and 274 DEGs were upregulated genes (Figure 1a and Table S1). And then, the prognostic genes in the TCGA–TNBC cohort were also identified. We extracted 48 overlapped genes as the prognostic HRD-associated genes (Figure 1b and Table S2). To further demonstrate how these 48 genes are involved in HRD, we performed GSEA and KEGG analysis for these 48 genes. The enrichment result indicated that these 48 genes might be involved in HRD through multiple pathways (Figure A1). Subsequently, patients in the TCGA–TNBC cohort were separated into the training set and the testing set randomly. No significant difference was found between the clinical features of two sets (Table S3). LASSO Cox method was used to construct a prognostic model based on the prognostic HRD-associated genes in the training set (Figure 1c and d). Finally, 16 genes were selected to construct the model (Table S4). And then, multivariate analysis was applied in the 16 selected genes on the prognosis in the TCGA–TNBC cohort. As shown in Figure 1e, ten genes (ZNF157, ST6GALNAC2, QRFPR, PEG10, OR2AE1, GSG1, GRM5, GAS2, FGL1, CSAG2, and ASCBG1) were protective factor for the prognosis in TNBC with hazard ratios (HRs) <1, while six genes (OTOR, NEUROD2, HOXD3, GSG1, EFNA5, and ACTL6B) were poor factors for the prognosis in TNBC with HR >1. Besides, 16 selected genes were independent prognostic factors in the TCGA–TNBC cohort (all *P* < 0.05). Finally, a signature based on the 16 selected genes was established and the risk scores of each patient were calculated by the following formula:

\begin{array}{c}\text{Risk}\hspace{.25em}\text{score}\\ \hspace{1em}=(-0.06442\times \text{expression}\hspace{.25em}\text{level}\hspace{.25em}\text{of ST6GALNAC2)}\\ \hspace{2em}+(0.1446\times \text{expression}\hspace{.25em}\text{level}\hspace{.25em}\text{of}\hspace{.25em}\text{ACTL6B})\\ \hspace{2em}+(-0.5976\times \text{expression}\hspace{.25em}\text{level}\hspace{.25em}\text{of}\hspace{.25em}\text{ACSBG1)}\\ \hspace{2em}+(-0.1383\times \text{expression}\hspace{.25em}\text{level}\hspace{.25em}\text{of}\hspace{.25em}\text{FGL1)}\\ \hspace{2em}+(0.1119\times \text{expression}\hspace{.25em}\text{level}\hspace{.25em}\text{of}\hspace{.25em}\text{GSG1})\\ \hspace{2em}+(0.2909\times \text{expression}\hspace{.25em}\text{level}\hspace{.25em}\text{of}\hspace{.25em}\text{OTOR})\\ \hspace{2em}+(0.0495\times \text{expression}\hspace{.25em}\text{level}\hspace{.25em}\text{of}\hspace{.25em}\text{HOXD3})\\ \hspace{2em}+\text{(}-0.3849\times \text{expression}\hspace{.25em}\text{level}\hspace{.25em}\text{of}\hspace{.25em}\text{ZNF157)}\\ \hspace{2em}+(-0.3886\times \text{expression}\hspace{.25em}\text{level}\hspace{.25em}\text{of}\hspace{.25em}\text{GAS2)}\\ \hspace{2em}+(-0.1003\times \text{expression}\hspace{.25em}\text{level}\hspace{.25em}\text{of}\hspace{.25em}\text{GRM5)}\\ \hspace{2em}+(1.0429\times \text{expression}\hspace{.25em}\text{level}\hspace{.25em}\text{of}\hspace{.25em}\text{NEUROD2})\\ \hspace{2em}+(0.5409\times \text{expression}\hspace{.25em}\text{level}\hspace{.25em}\text{of}\hspace{.25em}\text{EFNA5})\\ \hspace{2em}+\text{(}-0.1033\times \text{expression}\hspace{.25em}\text{level}\hspace{.25em}\text{of}\hspace{.25em}\text{QRFPR)}\\ \hspace{2em}+\text{(}-0.2160\times \text{expression}\hspace{.25em}\text{level}\hspace{.25em}\text{of}\hspace{.25em}\text{PEG10)}\\ \hspace{2em}+\text{(}-0.0998\times \text{expression}\hspace{.25em}\text{level}\hspace{.25em}\text{of}\hspace{.25em}\text{OR2AE1)}\\ \hspace{2em}+\text{(}-0.8540\times \text{expression}\hspace{.25em}\text{level}\hspace{.25em}\text{of}\hspace{.25em}\text{CSAG2)}\end{array}]

### 3.2 The 16-gene model had good performance in the prediction of TNBC prognosis

**Figure 2:**
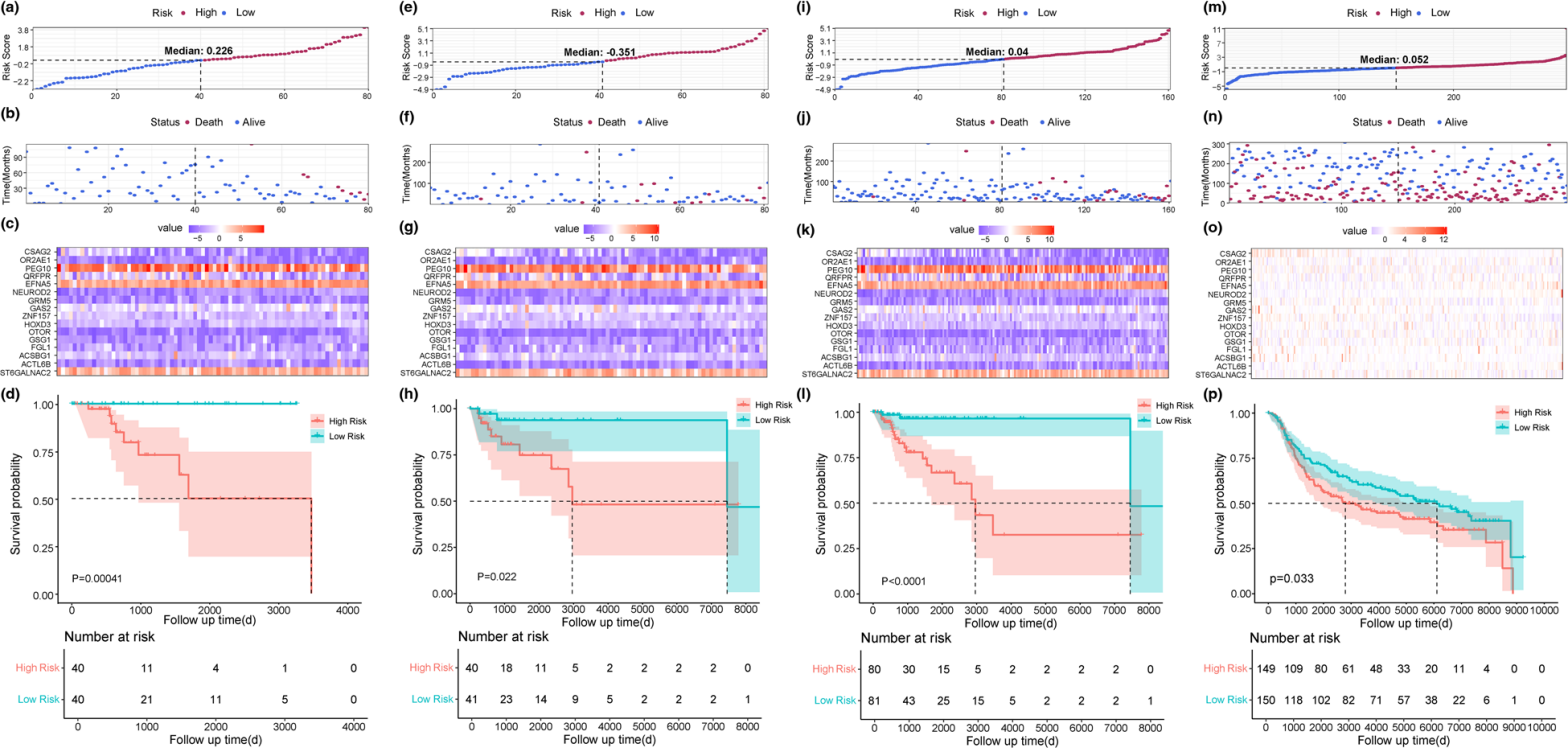
Evaluation and validation of the 16-gene model in TNBC. Distribution of risk score based on the 16-gene model in the training set (a), the testing set (e), the entire TCGA–TNBC cohort (i), and the METABRIC–TNBC cohort (m). Patterns of survival status and survival time of each patient in the training set (b), the testing set (f), the entire TCGA–TNBC cohort (j), and the METABRIC–TNBC cohort (n). Expression pattern of 16 genes of each patient in the training set (c), the testing set (g), the entire TCGA–TNBC cohort (k), and the METABRIC–TNBC cohort (o). Kaplan–Meier survival curves of the OS of patients in the high- and low-risk groups in the training set (d), the testing set (h), the entire TCGA–TNBC cohort (l), and the METABRIC–TNBC cohort (p).

Patients in the training set were divided into two groups (high risk and low risk) based on the median value of the risk score (Figure 2a, median value = 0.226). The status of each patient and the expression pattern of the 16 genes in each patient in the training set are shown in Figure 2b and c. Kaplan–Meier survival analysis showed that patients with high-risk score had worse OS than those with low-risk score (Figure 1d, *P* = 0.00041). As internal validation, patients in the testing set and the entire TCGA–TNBC cohort were also divided into high-risk and low-risk groups according to the median value of the risk score, respectively. The median value of the risk score in the testing set was −0.351 (Figure 2e) and in the entire TCGA–TNBC cohort was 0.04 (Figure 2i). The status of each patient and the expression pattern of the 16 genes in each patient in the training set are shown in Figure 2f and g, while those in the entire TCGA–TNBC cohort are shown in Figure 2j and k. Unsurprisingly, patients with TNBC with high-risk score also had inferior OS than those with low-risk score in both the testing set (Figure 2h, *P* = 0.022) and the entire TCGA–TNBC cohort (Figure 2l, *P* < 0.0001). As external validation, 299 patients with TNBC in the METABRIC dataset were enrolled in this study. The same processes were applied in the METABRIC cohort. Distribution of risk score, patterns of survival status and survival time of each patient, and the expression pattern of 16 genes in each patient are shown in Figure 2m–o. Besides, survival analysis showed that patients in the high-risk group had significantly shorter OS than those in the low-risk group (Figure 2p, *P* = 0.033).

**Figure 3:**
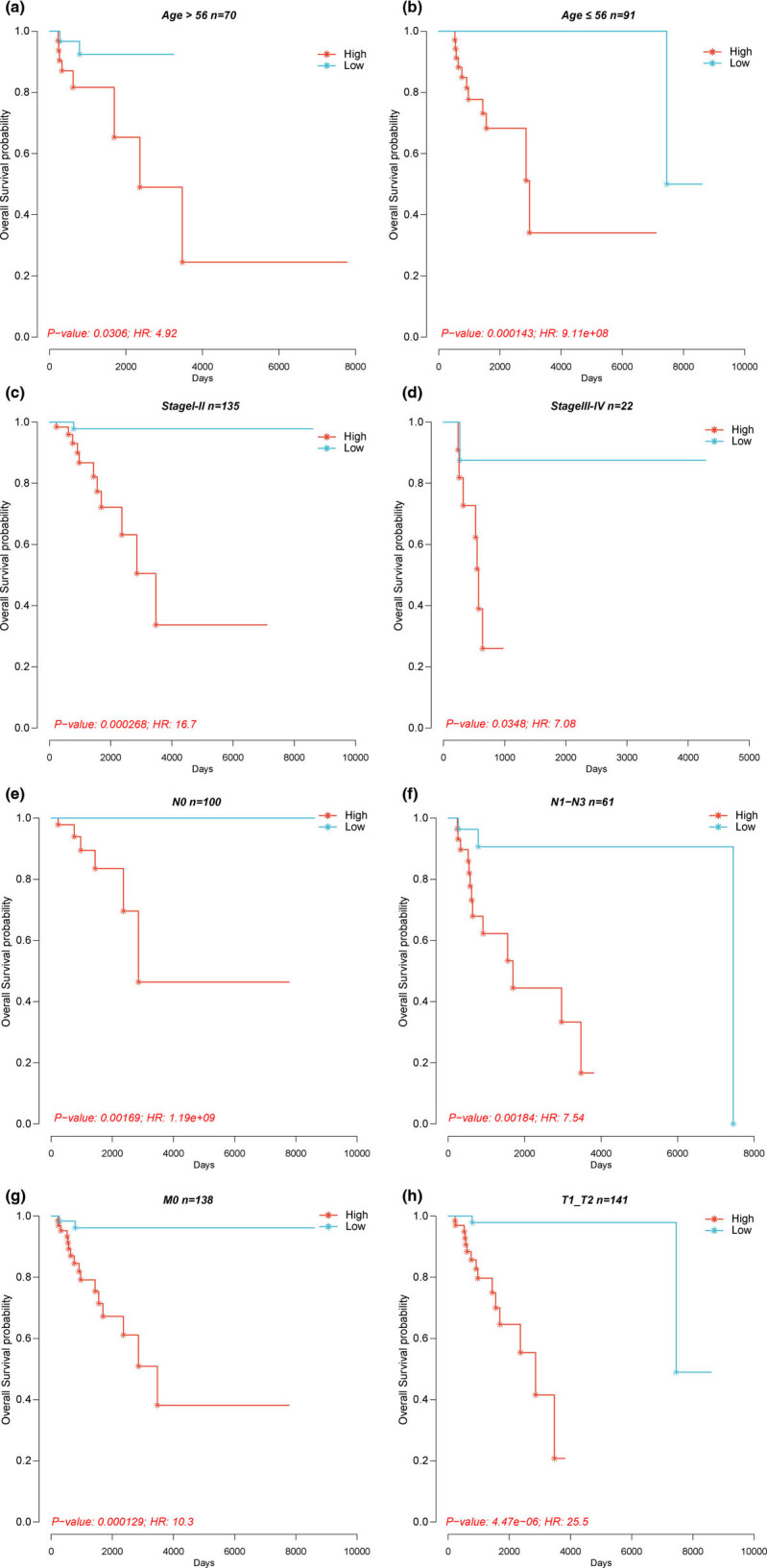
Kaplan–Meier survival analysis between the high-risk and low-risk groups in patients in the group of patients’ age older than 56 years (a), the group of patients’ age younger than 56 years (b), the group of patients with stages I–II (c), the group of patients with stages III–IV (d), the group of patients with N0 (e), the group of patients with N1–N3 (f), the group of patients with M0 (g), and the group of patients with T1–T2 (h).

**Figure A2:**
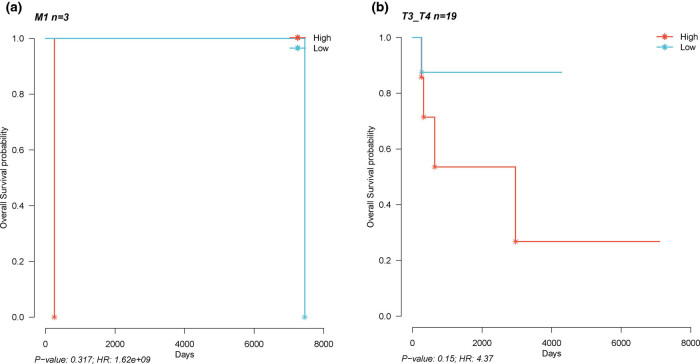
Kaplan–Meier survival analysis between high-risk and low-risk groups in patients in the group of patients with M1 (a) and the group of patients with T3–T4 (b).

**Figure A3:**
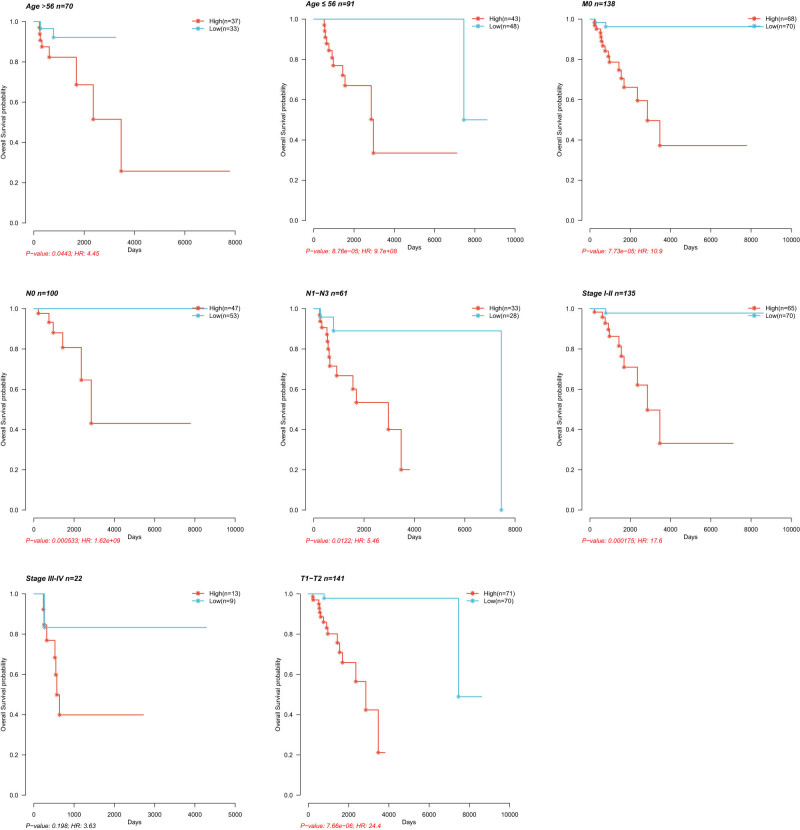
Kaplan–Meier survival analysis between high-risk and low-risk groups in patients in different subgroups.

To further investigate whether the prognostic value of the model can be impacted by the clinical characteristics, patients in the TCGA–TNBC cohort were stratified into different subgroups based on some clinical features including age ( <56 and ≥56 years), tumor stage (I + II and III + IV), T stage (T1 + T2 and T3 + T4), M stage (M0 and M1), and N stage (N0 and N1–N3). Subsequently, patients in each subgroup were further divided into the high-risk and low-risk groups based on the median value of the risk score. Unsurprisingly, the high-risk group was associated with poor prognosis in both the patients <56 years (Figure 3a, *P* = 0.0306) and ≥56 years (Figure 3b, *P* = 0.000143). Similarly, patients with high-risk score had worse OS than those with low-risk score no matter what tumor stage (Figure 3c, *P* = 0.000268; Figure 3d, *P* = 0.0348) and N stage (Figure 3e, *P* = 0.00169; Figure 3f, *P* = 0.00184) the patients are in. For patients in different T stage or M stage, the high-risk group was associated with inferior prognosis in the patients with M0 (Figure 3g, *P* = 0.000129) and in the patients with T1–T2 (Figure 3h, *P* = 4.47 × 10^−6^). However, no significant difference was found between the high-risk and low-risk groups in the patients with M1 (Figure A2a, *P* = 0.317) and in the patients with T3–T4 (Figure A2b, *P* = 0.15). The possible reason for this result was the small number of the patients with M1 (*n* = 3) and the patients with T3–T4 (*n* = 19). Moreover, we performed another procedure to improve the reliability of the result. The TCGA–TNBC cohort was first divided into the high-risk and low-risk groups, and the patients with particular clinical features were extracted from the two groups. The prognosis analysis was applied to compare the outcomes of the high-risk and low-risk groups, in particular clinical feature. Similarly, the high-risk score was associated with worse prognosis in all subgroups, except in the subgroup of patients with tumor stage III–IV (Figure A3).

**Figure A4:**
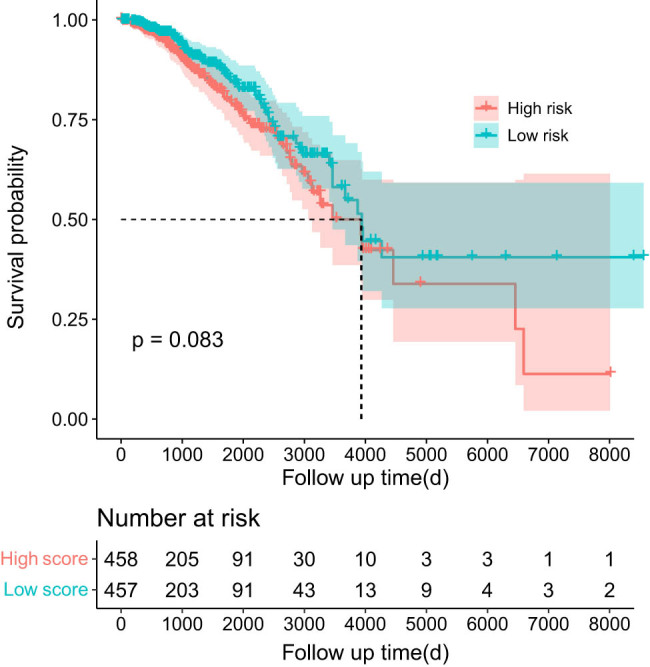
Prognosis analysis between high risk and low risk groups in the non-TNBC patients.

To further investigate whether our model only works in TNBC, we also calculated the risk score of patients with breast cancer with other subtype (non-TNBC) in the TCGA dataset and separated them into the high-risk group and the low-risk group based on the median value of the risk scores. And then, a comparison of the survival of patients between the high-risk and low-risk groups showed that no significant difference was found between the prognoses of the two groups, which indicated that our model failed to maintain a good predictive performance in non-TNBC patients (Figure A4, *P* = 0.083).

### 3.3 The 16-gene model had better predictive efficiency in TNBC

**Figure 4:**
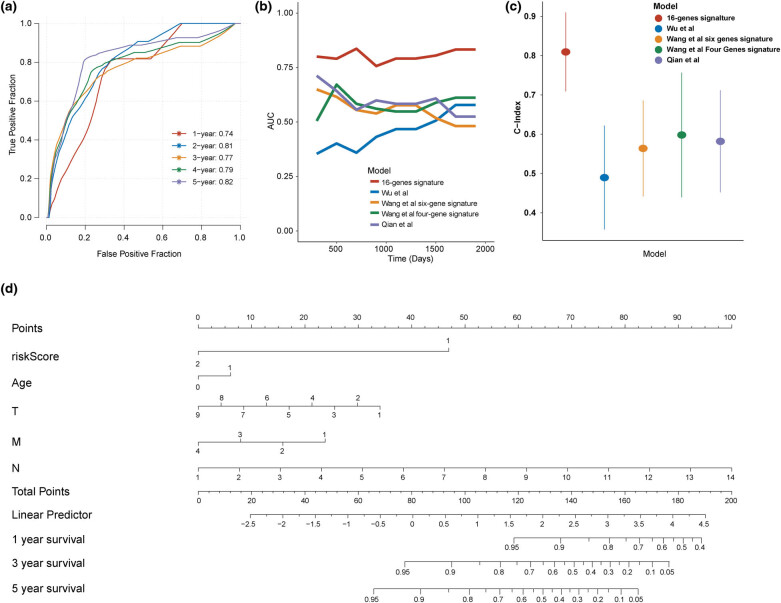
Evaluation of predictive efficiency of the 16-gene model in the TCGA–TNBC cohort. (a) Time-dependent ROC curve of 1, 2, 3, 4, and 5 years. (b) Comparison of the value of AUC between the 16-gene model and other four published models. (c) Comparison of the value of *C*-index between the 16-gene model and other four published models. (d) A nomogram based on the 16-gene model in TNBC.

The Univariate and multivariate analyses were performed in the TCGA–TNBC cohort, and multiple indexes were enrolled, including risk score based on the 16-gene signature (high risk vs low risk), age (≥56 and <56 years), tumor stage (III + IV vs I + II), and TMN stages. As shown in Table 1, our model was independent factor on the prognosis in TNBC (*P* = 0.004) while the N stage was the independent factor on the prognosis in TNBC as well (*P* = 0.0001). And then, the accuracy of the model in the prediction of 1-, 2-, 3-, 4-, and 5-year was evaluated by AUC. The values of AUC in 1-, 2-, 3-, 4-, and 5-year were 0.74, 0.81, 0.77, 0.79, and 0.82, respectively (Figure 4a). Subsequently, we collected four recently reported gene models in breast cancer, including a seven-nuclear receptor-based prognostic signature reported by Wu et al. [23], a six-gene signature associated with tumor mutation burden reported by Wang et al. [24], a four-gene signature in the tumor microenvironment reported by Wang et al [25], and a 17-gene signature reported by Qian et al. [26]. A comparison of AUC value and *C*-index value between the 16-gene model and other four reported models suggested that our model had better predictive efficiency in TNBC compared to other models (Figure 4b and c). Finally, we constructed the nomogram for the prediction of 1-, 3-, and 5-year survival in TNBC (Figure 4d).

**Table 1:** Univariate analysis and multivariate analysis of prognostic factors

Variables	Univariate analysis			Multivariate analysis		
	HR	95% CI	*P*-value^#^	HR	95% CI	*P*-value^#^
Risk score (high risk vs low risk)	0.117	0.035–0.399	0.001^**^	0.146	0.040–0.534	0.004^**^
Age (≥56 vs <56)	1.421	0.61–3.31	0.416	1.281	0.515–3.183	0.594
Tumor stage (III + IV vs I + II)	1.275	1.038–1.565	0.021*	1.009	0.767–1.327	0.949
T stage (T3–T4 vs T1–T2)	1.078	0.794–1.463	0.631	0.84	0.588–1.200	0.337
M stage (M1 vs M0)	1.252	0.331–2.371	0.49	0.722	0.318–1.640	0.437
N stage (N1–N3 vs N0)	1.382	1.225–1.559	0.00001^***^	1.371	1.179–1.595	0.0001^***^

HR: hazard ratio; CI: confidence interval.

^#^Chi-square test.

^*^ *P* < 0.05; ^**^ *P* < 0.01; ^***^ *P* < 0.001.

### 3.4 The 16-gene model was associated with immune signaling pathways in TNBC

**Figure 5:**
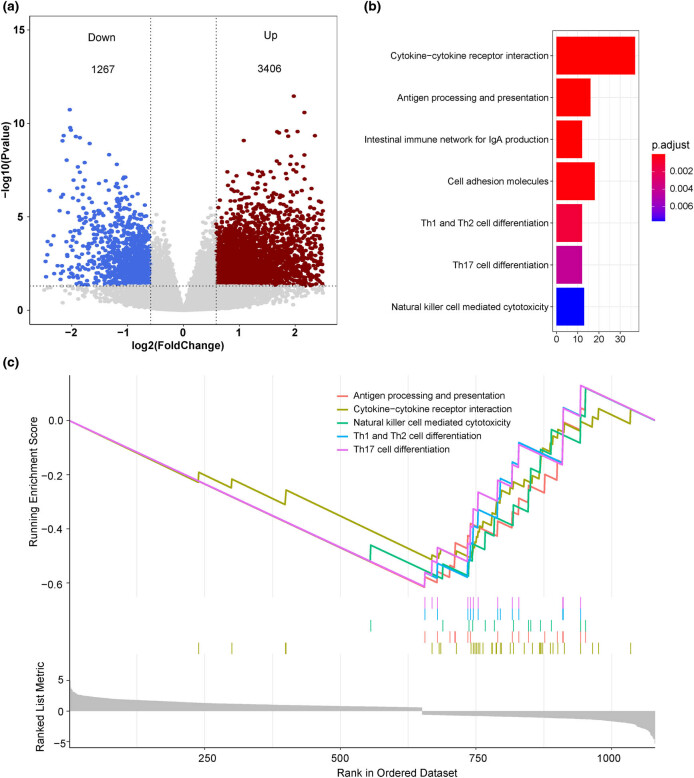
Mechanism exploration of the 16-gene model in TNBC. (a) Volcano plot of DEGs between the high-risk and low-risk groups in the TCGA–TNBC cohort. (b) KEGG pathway analysis of the DEGs. (c) GSEA of the DEGs.

**Figure A5:**
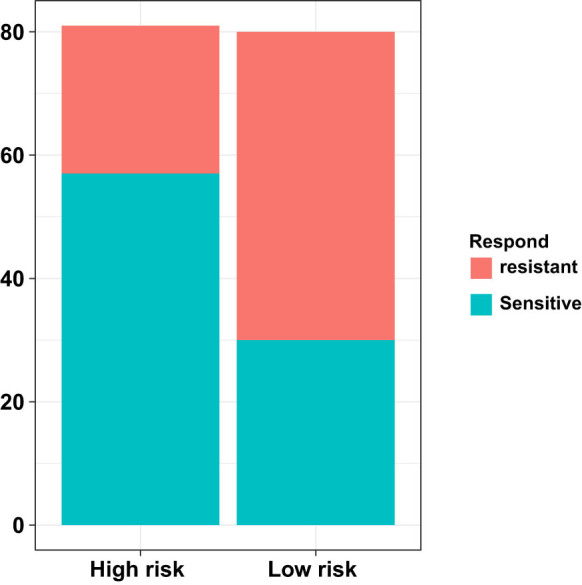
Immunotherapy response between high risk and low risk patients in the TCGA-TNBC cohort.

DEG analysis was performed between the high-risk and low-risk groups in the TCGA–TNBC cohort. A total of 1,267 downregulated genes and 3,406 upregulated genes were filtered as the DEGs (Figure 5a, Table S5). KEGG pathway analysis showed that the DEGs were enriched in the cytokine–cytokine receptor interaction, antigen processing and presentation, intestinal immune network for IgA production, cell adhesion molecules, Th1- and Th2-cell differentiation, Th17-cell differentiation, and natural killer cell-mediated cytotoxicity signaling pathways (Figure 5b). Moreover, GSEA showed that high-risk group was associated with inactivation of multiple immune-related signaling pathways including antigen processing and presentation, cytokine–cytokine receptor interaction, natural killer cell-mediated cytotoxicity, Th17 cell differentiation, and Th1 and Th2 cell differentiation (Figure 5c and Table S6). Besides, we predicted the immunotherapy response of patients in the TCGA–TNBC cohort by using the TIDE online tool [27] and compared the immunotherapy response between the high-risk and low-risk groups in the TCGA–TNBC cohort. The result showed that patients with high-risk scores were more sensitive to immunotherapy compared to those with low-risk scores (Figure A5).

## 4 Discussion

TNBC is a particularly aggressive subtype, accounting for approximately 15% of all breast cancers, known for its extremely poor prognosis. Recently, some advanced therapies have been developed including PARP-targeted therapy. Some PARP inhibitors, such as olaparib and talazoparib, have been approved to be used in the treatment of breast cancer [28,29]. For patients with the deficient of DNA double-strand damage repair (HRD), PARP inhibitor can further hinder the possibility of DNA repair in tumor cells, resulting in accelerating the death of tumor cells and implementing the precise targeting [15]. Hence, our aim is to establish a prognostic signature based on the HRD-associated genes in TNBC, expecting to provide a new strategy for risk stratification and precision treatment in TNBC. In this study, the HRD scores of 161 patients with TNBC in the TCGA cohort were calculated and the patients were divided into the high-HRD and low-HRD groups based on the median value of the HRD score. And then, DEG analysis and prognosis analysis were performed in two groups. Forty-eight genes were identified as the prognostic HRD-associated genes for model construction. The TCGA–TNBC cohort was divided into the training set and the testing set randomly. Subsequently, the LASSO method was used to establish a 16-gene prognostic model in the training set. Among these 16 genes, *ST6GALNAC2* has been reported to be the metastasis suppressor in breast cancer [30,31]. *FGL1* has been identified as the next immune checkpoint target [32] and dual-targeting FGL1/PD-L1 exhibited high synergistic therapeutic efficacy against breast cancer, even against TNBC [33,34]. Besides, some studies have demonstrated that *OTOR*, *HOXD3*, and *PEG10* were associated with prognosis in breast cancer [35,36,37].

To evaluate the robustness of our model, the risk score of each patient was calculated by a unified formula and the patients were separated into the high-risk and low-risk groups based on the median value of the risk scores. The high-risk group was associated with inferior prognosis in the training set, the testing set, and the entire TCGA–TNBC cohort. Besides, an external cohort with 299 patients with TNBC enrolled to validate the prognostic value of our model. Unsurprisingly, patients with high-risk score also had worse OS than those with low-risk score in the external cohort. In addition, ROC analysis and multivariate analysis also indicated that our model had great accuracy and independence in the prediction of prognosis for patients with TNBC. Besides, comparison of AUC value and *C*-index between our model and other four reported models further suggested that our model had better predictive efficiency compared to other published models in TNBC. Finally, a nomogram for 1-, 3-, and 5-year survival prediction in TNBC was constructed, except to improve the possibility of clinical application of our model.

To explore the potential mechanism of our model in TNBC, the TCGA–TNBC cohort was divided into the high-risk and low-risk groups based on the median value of the risk score. DEG analysis identified 4,673 DEGs between two groups. And then, we conducted the functional enrichment analysis in these DEGs. Notably, the DEGs were majorly enriched in some immune-related pathways, including antigen processing and presentation, cytokine–cytokine receptor interaction, natural killer cell-mediated cytotoxicity, Th17-cell differentiation, and Th1- and Th2-cell differentiation. Besides, the high-risk group was associated with the inactivation of these pathways. It is well-acknowledged that these pathways played crucial role in immunoregulation. For example, antigen processing and presentation is a complex process that involves in the identification of T-cell tumor antigens [38,39]. Th1, Th2, and Th17 are subgroups of CD4+ T cells, which play an important role in immunoregulation [40], and natural killer cells are powerful effectors of innate immunity that constitute a first line of defense against cancer as well [41]. Inactivation of these pathways can suppress the immune function in tumors, which can help tumor cells achieve immune escape. Our findings suggested that the poor prognosis of patients with high-risk score might be associated with immune evasion in TNBC. This result also indicated that our model had the potential to be the immunotherapy target in TNBC.

Even so, there are still some limitations in this study. For example, our model is more suitable for retrospective analysis and is still not clinically actionable. Besides, a larger sample size study is preferable. In the future, we will attempt to overcome these shortcomings by experiments.

## 5 Conclusion

We established a 16-gene prognostic signature based on the HRD score in TNBC, which had great performance in the prediction of prognosis and better predictive efficiency compared to other published models. Besides, we found that the risk score was associated with immunosuppression in TNBC. Finally, a nomogram based on our model was established. Our findings provided a new strategy for risk management in TNBC, and we expected to provide new thought for precision treatment in TNBC.

## Abbreviations

HRD: homologous recombination deficiency
TNBC: triple-negative breast cancer
ER: estrogen receptor
PR: progesterone receptor
HER-2: human epidermal growth factor receptor 2
PARP: poly-ADP-ribose polymerase
TCGA: The Cancer Genome Atlas
LASSO: Least absolute shrinkage and selection operator
OS: overall survival
ROC: receiver operating characteristic
AUC: area under curve
DEG: differentially expressed gene
KEGG: Kyoto Encyclopedia of Genes and Genomes
GSEA: Gene Set Enrichment Analysis

## Supplementary Material

Supplementary Table
